# Supplementary material for: Cis and trans RET signaling control the survival and central projection growth of rapidly adapting mechanoreceptors
Source: eLife. 2015 Apr 2;4:e06828. doi: 10.7554/eLife.06828 (PMC4408446; doi:10.7554/eLife.06828)
Supplement: Figure 1—source data 1. — DOI: http://dx.doi.org/10.7554/eLife.06828.004 [file elife06828s001.docx]

**Figure 1-source data 1: VGLUT1 dorsal spinal cord staining and RA mechanoreceptor number in P7 *Gfra2* mutants**

| Control genotype | VGluT1^+^ pixels  (% of control) | Mutant genotype | VGluT1^+^ pixels  (% of control) | P-value |
| --- | --- | --- | --- | --- |
| *Gfra2^GFP/+^* | 100±9.12 | *Gfra2^GFP/GFP^* | 96.31±8.97 | 0.96 |

#Thoracic SC only

| Control genotype | GFP^+^;NF200^+^ neurons/ DRG section | Mutant genotype | GFP^+^;NF200^+^ neurons/ DRG section | P-value |
| --- | --- | --- | --- | --- |
| *Gfra2^GFP/+^* | 7.13±0.48 | *Gfra2^GFP/GFP^* | 6.50±0.41 | 0.34 |

#L4/L5 DRGs only
