# Supplementary material for: Cis and trans RET signaling control the survival and central projection growth of rapidly adapting mechanoreceptors
Source: eLife. 2015 Apr 2;4:e06828. doi: 10.7554/eLife.06828 (PMC4408446; doi:10.7554/eLife.06828)
Supplement: Figure 1—source data 2. — DOI: http://dx.doi.org/10.7554/eLife.06828.005 [file elife06828s002.docx]

**Figure 1-source data 2: RA mechanoreceptor central projections and cell number in E13.5 *Ret, Gfra1, Gfra2,* and *Nrtn* mutants**

| **SC Level** | **Control genotype** | **GFP^+^ pixels (% of control)** | **Mutant genotype** | **GFP^+^ pixels (% of control)** | **P-value** |
| --- | --- | --- | --- | --- | --- |
| Cervical | *Ret^CFP/+^* | 100±13.61 | *Ret^CFP/CFP^* | 28.12±12.25 | <0.0001 |
| Thoracic | *Ret^CFP/+^* | 100±9.18 | *Ret^CFP/CFP^* | 42.88±8.93 | 0.0001 |
| Lumbar | *Ret^CFP/+^* | 100±8.96 | *Ret^CFP/CFP^* | 37.78±6.84 | <0.0001 |
| Cervical | *Gfra1^+/-^; Ret^CFP/+^* | 100±6.85 | *Gfra1^-/-^;Ret^CFP/+^* * | 143.33±15.92 | 0.02 |
| Thoracic | *Gfra1^+/-^; Ret^CFP/+^* | 100±9.68 | *Gfra1^-/-^;Ret^CFP/+^* * | 134.65±14.14 | 0.05 |
| Lumbar | *Gfra1^+/-^; Ret^CFP/+^* | 100±9.76 | *Gfra1^-/-^;Ret^CFP/+^* * | 128.01±11.31 | 0.07 |
| Cervical | *Gfra2^+/-^;Ret^CFP/+^* | 100±13.42 | *Gfra2^-/-^;Ret^CFP/+^* | 12.78±1.86 | <0.0001 |
| Thoracic | *Gfra2^+/-^;Ret^CFP/+^* | 100±16.95 | *Gfra2^-/-^;Ret^CFP/+^* | 9.50±1.44 | <0.0001 |
| Lumbar | *Gfra2^+/-^;Ret^CFP/+^* | 100±14.52 | *Gfra2^-/-^;Ret^CFP/+^* | 5.57±0.77 | <0.0001 |
| Cervical | *Nrtn^+/-^;Ret^CFP/+^* | 100±13.43 | *Nrtn^-/-^;Ret^CFP/+^* | 47.95±8.23 | 0.0002 |
| Thoracic | *Nrtn^+/-^;Ret^CFP/+^* | 100±13.64 | *Nrtn^-/-^;Ret^CFP/+^* | 26.47±3.84 | <0.0001 |
| Lumbar | *Nrtn^+/-^;Ret^CFP/+^* | 100±14.12 | *Nrtn^-/-^;Ret^CFP/+^* | 43.47±6.49 | 0.002 |

*The slight increase of CFP^+^ signal in *Gfra1* mutant mice could be due to the precocious appearance of *Ret^+^* dSC cells, which are also stained by anti-GFP antibody.

| **DRG Level** | **Control genotype** | **GFP^+^ neurons per DRG section** | **Mutant genotype** | **GFP^+^ neurons per DRG section** | **P-value** |
| --- | --- | --- | --- | --- | --- |
| Cervical | *Ret^CFP/+^* | 25.81±1.74 | *Ret^CFP/CFP^* | 23.44±2.03 | 0.38 |
| Thoracic | *Ret^CFP/+^* | 19.94±1.42 | *Ret^CFP/CFP^* | 18.88±1.50 | 0.61 |
| Lumbar | *Ret^CFP/+^* | 21.81±1.29 | *Ret^CFP/CFP^* | 17.16±2.01 | 0.06 |
| Cervical | *Gfra1^+/-^;Ret^CFP/+^* | 24.89±1.82 | *Gfra1^-/-^;Ret^CFP/+^* | 23.38±3.51 | 0.39 |
| Thoracic | *Gfra1^+/-^;Ret^CFP/+^* | 19.43±2.02 | *Gfra1^-/-^;Ret^CFP/+^* | 19.00±1.56 | 0.87 |
| Lumbar | *Gfra1^+/-^;Ret^CFP/+^* | 20.27±1.24 | *Gfra1^-/-^;Ret^CFP/+^* | 23.17±1.20 | 0.11 |
| Cervical | *Gfra2^+/-^;Ret^CFP/+^* | 40.38±2.97 | *Gfra2^-/-^;Ret^CFP/+^* | 36.57±3.53 | 0.41 |
| Thoracic | *Gfra2^+/-^;Ret^CFP/+^* | 30.94±1.21 | *Gfra2^-/-^;Ret^CFP/+^* | 27.67±1.69 | 0.13 |
| Lumbar | *Gfra2^+/-^;Ret^CFP/+^* | 28.29±1.39 | *Gfra2^-/-^;Ret^CFP/+^* | 24.59±1.53 | 0.09 |
| Cervical | *Nrtn^+/-^;Ret^CFP/+^* | 42.93±3.00 | *Nrtn^-/-^;Ret^CFP/+^* | 44.50±3.22 | 0.72 |
| Thoracic | *Nrtn^+/-^;Ret^CFP/+^* | 30.38±1.14 | *Nrtn^-/-^;Ret^CFP/+^* | 28.56±1.75 | 0.39 |
| Lumbar | *Nrtn^+/-^;Ret^CFP/+^* | 27.31±1.29 | *Nrtn^-/-^;Ret^CFP/+^* | 25.00±1.45 | 0.24 |
