# Supplementary material for: Cis and trans RET signaling control the survival and central projection growth of rapidly adapting mechanoreceptors
Source: eLife. 2015 Apr 2;4:e06828. doi: 10.7554/eLife.06828 (PMC4408446; doi:10.7554/eLife.06828)
Supplement: Figure 3—source data 1. — DOI: http://dx.doi.org/10.7554/eLife.06828.011 [file elife06828s003.docx]

**Figure 3-source data 1: RA mechanoreceptor central projections and cell number in E15.5 *Gfra2* mutants**

| Control genotype | Tdt^+^ dSC pixels (% of control) | Mutant genotype | Tdt^+^ dSC pixels (% of control) | P-value |
| --- | --- | --- | --- | --- |
| *Gfra2^GFP/+^; Ret^CreERT/+^;Rosa^Tdt^* | 100±8.80 | *Gfra2^GFP/GFP^; Ret^CreERT/+^;Rosa^Tdt^* | 55.13±2.82 | <0.0001 |

# Thoracic spinal cord only

| Control genotype | Tdt^+^  neurons per DRG section (% of control) | Mutant genotype | Tdt^+^  neurons per DRG section (% of control) | P-value |
| --- | --- | --- | --- | --- |
| *Gfra2^GFP/+^; Ret^CreERT/+^;Rosa^Tdt^* | 100±6.32 | *Gfra2^GFP/GFP^; Ret^CreERT/+^;Rosa^Tdt^* | 79.52±8.39 | 0.06 |

#L4/L5 DRGs only
