# Supplementary material for: Cis and trans RET signaling control the survival and central projection growth of rapidly adapting mechanoreceptors
Source: eLife. 2015 Apr 2;4:e06828. doi: 10.7554/eLife.06828 (PMC4408446; doi:10.7554/eLife.06828)
Supplement: Figure 4—source data 1. — DOI: http://dx.doi.org/10.7554/eLife.06828.014 [file elife06828s004.docx]

**Figure 4-source data 1: RA mechanoreceptor central projections and cell number in E18.5 *Ret*, *Gfra2*, *Gfra1*, and *Gfra1;Gfra2* mutants**

| Control genotype | Tdt^+^ dSC pixels (% of control) | Mutant genotype | Tdt^+^ dSC pixels (% of control) | P-value |
| --- | --- | --- | --- | --- |
| *Ret^CreERT/+^;*  *Rosa^Tdt^* | 100±9.82 | *Ret^CreERT/CreERT^;*  *Rosa^Tdt^* | 35.86±4.97 | <0.0001 |
| *Gfra2^GFP/+^; Ret^CreERT/+^;Rosa^Tdt^* | 100±2.53 | *Gfra2^GFP/GFP^; Ret^CreERT/+^;Rosa^Tdt^* | 86.34±4.48 | 0.01 |
| *Gfra1^+/-^; Ret^CreERT/+^;Rosa^Tdt^* | 100±2.14 | *Gfra1^-/-^; Ret^CreERT/+^;Rosa^Tdt^* | 80.94±10.32 | 0.09 |
| *Gfra1^+/-^; Gfra2^GFP/+^; Ret^CreERT/+^;Rosa^Tdt^* | 100±4.49 | *Gfra1^-/-^; Gfra2^GFP/GFP^; Ret^CreERT/+^;Rosa^Tdt^* | 27.25±2.09 | <0.0001 |

# Thoracic spinal cord only

| Control genotype | Tdt^+^  neurons per DRG (% of control) | Mutant genotype | Tdt^+^  neurons per DRG (% of control) | P-value |
| --- | --- | --- | --- | --- |
| *Ret^CreERT/+^;Rosa^Tdt^* | 100±8.71 | *Ret^CreERT/CreERT^;Rosa^Tdt^* | 52.52±7.76 | 0.0007 |
| *Gfra2^GFP/+^; Ret^CreERT/+^;Rosa^Tdt^* | 100±5.10 | *Gfra2^GFP/GFP^; Ret^CreERT/+^;Rosa^Tdt^* | 84.01±5.16 | 0.04 |
| *Gfra1^+/-^; Ret^CreERT/+^;Rosa^Tdt^* | 100±6.72 | *Gfra1^-/-^; Ret^CreERT/+^;Rosa^Tdt^* | 82.30±12.91 | 0.19 |
| *Gfra1^+/-^; Gfra2^GFP/+^; Ret^CreERT/+^;Rosa^Tdt^* | 100±8.61 | *Gfra1^-/-^; Gfra2^GFP/GFP^; Ret^CreERT/+^;Rosa^Tdt^* | 38.17±2.65 | 0.0002 |

#L4/L5 DRGs only
