# Supplementary material for: Cis and trans RET signaling control the survival and central projection growth of rapidly adapting mechanoreceptors
Source: eLife. 2015 Apr 2;4:e06828. doi: 10.7554/eLife.06828 (PMC4408446; doi:10.7554/eLife.06828)
Supplement: Figure 6—source data 1. — DOI: http://dx.doi.org/10.7554/eLife.06828.018 [file elife06828s005.docx]

| **Age** | **Genotype** | **ΔCT (*Gfra1-Gapdh)*** | **ΔCT S.D.** | **Relative expression normalized to E13.5 *Gfra2^-/-^* (2^-ΔΔCT^)** |
| --- | --- | --- | --- | --- |
| E13.5 | *Gfra2^+/-^* | 6.3449 | 0.2873 | 0.819-1.220 |
| E13.5 | *Gfra2^-/-^* | 6.4359 | 0.1977 | 0.819-1.077 |
| E15.5 | *Gfra2^+/-^* | 6.9424 | 0.2094 | 0.572-0.764 |
| E15.5 | *Gfra2^-/-^* | 6.8770 | 0.1723 | 0.614-0.779 |
| E18.5 | *Gfra2^+/-^* | 6.0577 | 0.1759 | 1.080-1.379 |
| E18.5 | *Gfra2^-/-^* | 6.2467 | 0.1935 | 0.939-1.228 |

**Figure 6-source data 1: QPCR of *Gfra1* in embryonic *Gfra2* null DRGs**

| **Population 1** | **Population 2** | **P-value** |
| --- | --- | --- |
| E13.5 *Gfra2^+/-^* | E13.5 *Gfra2^-/-^* | 0.675 |
| E15.5 *Gfra2^+/-^* | E15.5 *Gfra2^-/-^* | 0.698 |
| E18.5 *Gfra2^+/-^* | E18.5 *Gfra2^-/-^* | 0.288 |
