# Supplementary material for: Cis and trans RET signaling control the survival and central projection growth of rapidly adapting mechanoreceptors
Source: eLife. 2015 Apr 2;4:e06828. doi: 10.7554/eLife.06828 (PMC4408446; doi:10.7554/eLife.06828)
Supplement: Figure 7—source data 1. — DOI: http://dx.doi.org/10.7554/eLife.06828.021 [file elife06828s006.docx]

**Figure 7-source data 1: Quantification of axonal growth in *Ret* mutant DRG explants**

| Treatment | Control genotype | Axon intersections at 200μm | Mutant genotype | Axon intersections at 200μm | p-value |
| --- | --- | --- | --- | --- | --- |
| NRTN | *Ret^CFP/+^* | 147.833±10.579 (n=6) | *Ret^CFP/CFP^* | 0 (n=8) | <0.0001 |
| GDNF | *Ret^CFP/+^* | 152.500±21.165 (n=8) | *Ret^CFP/CFP^* | 0.875±0.543 (n=8) | <0.0001 |
| GDNF+ GFRa1 | *Ret^CFP/+^* | 165.375±18.029 (n=8) | *Ret^CFP/CFP^* | 0.375±0.246 (n=8) | <0.0001 |
| GFRa1 | *Ret^CFP/+^* | 7.428±1.104 (n=7) | *Ret^CFP/CFP^* | 1.875±0.695 (n=8) | 0.0002 |
