# Supplementary material for: Cis and trans RET signaling control the survival and central projection growth of rapidly adapting mechanoreceptors
Source: eLife. 2015 Apr 2;4:e06828. doi: 10.7554/eLife.06828 (PMC4408446; doi:10.7554/eLife.06828)
Supplement: Figure 7—source data 2. — DOI: http://dx.doi.org/10.7554/eLife.06828.022 [file elife06828s007.docx]

**Figure 7-source data 2: GFP^+^ neuron number in *Gfra2* null and *Gfra2;Ntrk1* double null explants**

| Treatment | Control genotype | GFP^+^ neurons/10,000μm^2^ | Mutant genotype | GFP^+^ neurons/10,000μm^2^ | p-value |
| --- | --- | --- | --- | --- | --- |
| NRTN | *Ret^CFP/+^* | 4.825±0.545 (n=6) | *Ret^CFP/CFP^* | 0.052±0.036 (n=8) | <0.0001 |
| GDNF | *Ret^CFP/+^* | 4.917±0.619 (n=8) | *Ret^CFP/CFP^* | 0.045±0.022 (n=8) | <0.0001 |
| GDNF + GFRa1 | *Ret^CFP/+^* | 4.776±0.539 (n=8) | *Ret^CFP/CFP^* | 0.062±0.041 (n=8) | <0.0001 |
| GFRa1 | *Ret^CFP/+^* | 0.278±0.077 (n=7) | *Ret^CFP/CFP^* | 0.075±0.038 (n=8) | 0.0245 |
| NRTN | *Gfra2^GFP/+^* | 2.394±0.344 (n=8) | *Gfra2^GFP/GFP^* | 0.464±0.176 (n=7) | <0.0001 |
| GDNF | *Gfra2^GFP/+^* | 3.061±0.401 (n=8) | *Gfra2^GFP/GFP^* | 2.968±0.554 (n=7) | 0.7513 |
| GDNF + GFRa1 | *Gfra2^GFP/+^* | 3.982±0.559 (n=7) | *Gfra2^GFP/GFP^* | 2.941±0.461 (n=6) | 0.1099 |
| GFRa1 | *Gfra2^GFP/+^* | 0.201±0.103 (n=6) | *Gfra2^GFP/GFP^* | 0.122±0.052 (n=6) | 0.3635 |
| NRTN | *Gfra2^GFP/+^; Ntrk1^-/-^* | 1.247±0.237 (n=12) | *Gfra2^GFP/GFP^ ; Ntrk1^-/-^* | 0 (n=6) | <0.0001 |
| GDNF | *Gfra2^GFP/+^; Ntrk1^-/-^* | 1.113±0.268 (n=10) | *Gfra2^GFP/GFP^ ; Ntrk1^-/-^* | 0.033±0.031 (n=10) | <0.0001 |
| GDNF + GFRa1 | *Gfra2^GFP/+^; Ntrk1^-/-^* | 3.381±0.522 (n=11) | *Gfra2^GFP/GFP^ ; Ntrk1^-/-^* | 0.389±0.144 (n=8) | <0.0001 |
| GFRa1 | *Gfra2^GFP/+^; Ntrk1^-/-^* | 0.020±0.019 (n=9) | *Gfra2^GFP/GFP^ ; Ntrk1^-/-^* | 0 (n=6) | <0.0001 |

| Genotype | Treatment | GFP^+^ neurons/10,000μm^2^ | Treatment | GFP^+^ neurons/10,000μm^2^ | p-value |
| --- | --- | --- | --- | --- | --- |
| *Gfra2^GFP/GFP^ ; Ntrk1^-/-^* | GDNF + GFRa1 | 0.389±0.144 (n=10) | GDNF | 0.033±0.031 (n=8) | 0.0011 |
