# Supplementary material for: Cis and trans RET signaling control the survival and central projection growth of rapidly adapting mechanoreceptors
Source: eLife. 2015 Apr 2;4:e06828. doi: 10.7554/eLife.06828 (PMC4408446; doi:10.7554/eLife.06828)
Supplement: Figure 7—source data 3. — DOI: http://dx.doi.org/10.7554/eLife.06828.023 [file elife06828s008.docx]

**Figure 7-source data 3: Densimetric measurements of GFRa1 in DRG cell extracts and supernatants**

| **Cell lysates** |  |  |
| --- | --- | --- |
| **Genotype** | **Band density (A.U.)** | **P-value (relative to wild type)** |
| Wild type | 1±0.131 | N/A |
| *Gfra2^-/-^* | 0.798±0.192 | 0.434 |
| *Gfra1^-/-^* | 0.024±0.007 | 0.002 |

| **Supernatants** |  |  |
| --- | --- | --- |
| **Genotype** | **Band density (A.U.)** | **P-value (relative to wild type)** |
| Wild type | 1±0.162 | N/A |
| *Gfra2^-/-^* | 0.873±0.116 | 0.556 |
| *Gfra1^-/-^* | 0.005±0.003 | 0.004 |

A.U.=arbitrary units
